# Remarks upon Trephining the Cranium

**Published:** 1895-07

**Authors:** John Ashhurst

**Affiliations:** Barton Professor of Surgery and Professor of Clinical Surgery in the University of Pennsylvania, Surgeon to the Pennsylvania Hospital, etc.


### REMARKS UPON TREPHINING THE CRANIUM.

BY JOHN ASHHURST, JR., M. D.,
Barton Professor of Surgery and Professor of Clinical Surgery in the University of Pennsylvania, Surgeon to the Pennsylvania Hospital, etc.

I find that I have performed the operation of trephining the skull forty-one times, not including those cases in which I have merely opened the frontal sinuses, nor those in which I have removed bone fragments without using the trephine. Of these forty-one cases twenty ended in recovery and twenty-one in death, showing a mortality of little more than 50 per cent. In many instances I have refrained from interference when other surgeons would have operated, so that my cases have been of an unfavorable type, and the mortality has no doubt been higher than if I had operated more indiscriminately.

The details of these cases are as follows: Twenty-four were primary operations for compound fracture, with eleven recoveries and thirteen deaths; two were operations during the intermediate period, both successful; and three were secondary operations, with one recovery and two deaths, both in cases of abscess.

As far as it goes this analysis confirms what has so often been pointed out, that there is not as much urgency in operating upon compound fractures of the skull as there is in compound fractures of the extremities. In the latter, the sooner the operation is done, if the patient is able to bear it, the better. This has long been the rule in military surgery, when amputation is required, and some years ago I collected extensive statistics from civil practice which showed that the same rule of procedure applied there. But this is not so in compound fractures of the skull, and the proportion of recoveries is larger in delayed cases than when the operation is done immediately, as is well shown by Bluhm's statistics. At the same time, in a bad case, where an operation is evidently necessary, I do not advise delay; but early trephining is not so imperative as in early amputation for compound fractures of the long bones. Trephining for suppuration, occurring as the result of injury, is usually fatal.

In three cases I have operated for syphilitic disease, with two deaths and one recovery. In the latter case, besides evidence of syphilitic brain disease, there were painful nodes on the skull, and I operated by dividing the nodes with a Hey's saw, and then made a single opening with the trephine, so as to relieve the intracranial tension. The patient was much benefited for a time, and left the hospital relieved, though not cured. The fatal cases were in patients suffering from syphilis of long standing, with necrosis and intracranial suppuration.

I have been induced to trephine in three cases of epilepsy, all the patients recovering from the operation. One, an epileptic with suicidal tendencies, came under my care at the University Hospital in October, 1886. After the operation the patient was much benefited as long as he remained under observation. In the other two there was no evident improvement, though both did well as regarded the operation. In a case of melancholia, following an old fracture of the skull, trephining gave no relief, and of two cases in which I have operated for convulsions, etc., following old injury, one terminated fatally, while no permanent gain resulted in the other.

I have operated unsuccessfully upon three patients for the cerebral complications resulting from disease of the middle ear. Statistics show that many lives have been saved by trephining under these circumstances, but in my own cases, though the abscesses have been reached and evacuated, the patients have died.

Although I have thus operated in twenty-one fatal cases by trephining, in only one case did the operation seem to have been responsible for the patient's death. This case was that of a child with a depressed fracture over the lateral sinus. On removing the depressed bone, profuse hemorrhage occurred, and the patient died in consequence. I had not then learned the futility of attempting to check bleeding from the brain sinuses, except by prompt plugging. I have had four cases since in which the longitudinal sinus was opened, and in two of them the patient recovered. In a third, bleeding was readily controlled by pressure, but ultimately death followed; while in the fourth a clot had formed in the sinus, giving time to apply a lateral suture to the divided vessel. This case was an interesting one; it was that of a boy who had been injured by a nitro-glycerin explosion, a piece of the metal being found lodged in the longitudinal sinus, causing a clot as mentioned.

As regards the locality of the injury, I find that of fractures involving the frontal bone, omitting those simply involving the frontal sinuses, there were five, with four recoveries and one death. These figures do not confirm the general impression that there is special danger in fractures of the frontal bone. Indeed, much more depends upon the amount of injury to the brain than upon the place of the fracture. In one case the indication for trephining was bleeding from the middle meningeal artery, and in that case the patient recovered. He was an athlete, who, while playing football, came into violent collision with another player, sustaining a fissured fracture of the parietal bone. He was stunned at the time, but soon recovered consciousness; in the course of half an hour, however, convulsions came on, followed by coma. He was brought to the hospital, and I applied the trephine, evacuating a considerable quantity of clot; the patient made an uninterrupted recovery.

DISCUSSION.

DR. H. R. WHARTON: I remember seeing the case of the boy who died of hemorrhage during the operation, that Dr. Ashhurst referred to, and I assisted him in the operation. As soon as the fractured bone was elevated there was a great gush of blood and the patient died in a few minutes. I am sorry that Dr. Ashhurst did not say anything about fractures of the base of the skull. I would like to have his views as to the use of the trephine in this class of cases.

DR. R. H. HARTE: I would like to ask, with regard to fractures of the base, if any of the Fellows have seen cases of this kind with escape of cerebral substance from the ears. I have seen two cases in both of which recovery followed. Some years ago a man was brought into the Pennsylvania Hospital with fractured skull and brain substance oozing from the ear. For several months he seemed melancholic, but finally recovered with improved mental condition. The next case occurred only a few months ago. A child fell down stairs and was found unconscious with brain substance flowing from the ears. That child recovered without any alteration or impairment of the mental faculties.

THE PRESIDENT: I would ask Dr. Ashhurst, in cases where there is a marked depressed fracture of the skull, whether he would prefer to leave it and wait for developments, or would he, in his judgment, think it best to proceed at once.

In terminating the discussion, Dr. Ashhurst said: In reply to the last question, I wished to be understood as saying that I would not push the argument from statistics, and that in cases in which the operation was clearly indicated I would operate at once; but that in cases where there was a doubt in the mind of the surgeon as to whether he should operate or not, a short delay would not be as injurious as it would be in the case of amputation. In cases of impacted fracture my practice has been, as a rule, not to interfere in the absence of symptoms. In cases where there is no opening into the cranial cavity and where there are no cerebral symptoms, I think the surgeon is justified in waiting for more definite indications. At the same time I find myself more inclined to operate than I was twenty years ago, on account of the greater safety afforded by modern methods of wound treatment.

With regard to Dr. Harte's question, I do not recall any case of discharge of brain matter from the ear, but loss of brain substance not infrequently occurs at the seat of fracture. With regard to the watery discharge from the ear, I have often observed that as long as the discharge continues the patient may do well, but that if it suddenly stops the patient will probably become comatose and die in a few hours; the arrest of this flow seems to increase the pressure upon the brain. Old residents of the Pennsylvania Hospital will remember a case under the care of the late Professor Joseph Pancoast, in which there had been such a free discharge from the ear that it had been collected in a cup to be measured. The cup was found empty in the morning, and upon investigation it was learned that the night watchman had mistaken the fluid for medicine and had administered it to the patient, a tablespoonful every three hours, during the night.

With regard to fractures at the base of the skull, I have not seen any case which I thought trephining indicated. I think that an attempt should be made to prevent infection in these cases by cleansing the ears, and, as far as possible, the nasal and buccal cavities. After securing cleanliness I rely upon the use of calomel and Dover's powder, with hygienic treatment, rest in bed, cold to the head, laxative enemata, etc., and by these means recovery will be obtained in a considerable number of cases.

THE PRESIDENT.—In cases of compound fracture of the skull, with probably slight depression, I have always given the patient the benefit of the doubt. I think it is much safer to remove a disk of bone than to run any possible risk from depressed fracture. I should always prefer the early operation in these cases rather than to delay.

